# Growth differentiation factor 15 predicts poor prognosis in patients with heart failure and reduced ejection fraction and anemia: results from RED-HF

**DOI:** 10.1007/s00392-021-01944-6

**Published:** 2021-10-05

**Authors:** Thor Ueland, Lars Gullestad, Lei Kou, James B. Young, Marc A. Pfeffer, Dirk Jan van Veldhuisen, Karl Swedberg, John J. V. Mcmurray, Akshay S. Desai, Inderjit S. Anand, Pål Aukrust

**Affiliations:** 1 Research Institute of Internal Medicine, Oslo University Hospital, Rikshospitalet, Nydalen, P. B. 4950, 0424 Oslo, Norway; 2 Department of Cardiology, Oslo University Hospital, Rikshospitalet, Oslo, Norway; 3 Section of Clinical Immunology and Infectious Diseases, Oslo University Hospital, Rikshospitalet, Oslo, Norway; 4 Faculty of Medicine, University of Oslo, Oslo, Norway; 5 Center for Heart Failure Research, University of Oslo, Oslo, Norway; 6 K.G. Jebsen Cardiac Research Center, University of Oslo, Oslo, Norway; 7 K. G. Jebsen Inflammation Research Center, University of Oslo, Oslo, Norway; 8 K. G. Jebsen Thrombosis Research and Expertise Center, University of Tromsø, Tromsö, Norway; 9 Cleveland Clinic, Cleveland, OH USA; 10 Cardiovascular Division, Brigham and Women’s Hospital, Boston, MA USA; 11 VA Medical Center, Minneapolis, MN USA; 12 University of Minnesota, Minneapolis, MN USA; 13 University Medical Center Groningen, University of Groningen, Groningen, The Netherlands; 14 Sahlgrenska Academy, University of Gothenburg, Gothenburg, Sweden; 15 National Heart and Lung Institute, Imperial College, London, UK; 16 BHF Glasgow Cardiovascular Research Centre, University of Glasgow, Glasgow, UK

**Keywords:** GDF-15, Anemia, Heart failure, Prognosis

## Abstract

**Aims:**

We aimed to assess the value of GDF-15, a stress-responsive cytokine, in predicting clinical outcomes in patients with heart failure (HF) with reduced ejection fraction (HFrEF) and anemia

**Methods and results:**

Serum GDF-15 was assessed in 1582 HFrEF and mild-to-moderate anemia patients who where followed for 28 months in the Reduction of Events by Darbepoetin alfa in Heart Failure (RED-HF) trial, an overall neutral RCT evaluating the effect darbepoetin alfa on clinical outcomes in patients with systolic heart failure and mild-to-moderate anemia. Association between baseline and change in GDF-15 during 6 months follow-up *and* the primary composite outcome of all-cause death or HF hospitalization were evaluated in multivariable Cox-models adjusted for conventional clinical and biochemical risk factors. The adjusted risk for the primary outcome increased with (i) successive tertiles of baseline GDF-15 (tertile 3 HR 1.56 [1.23–1.98] *p* < 0.001) as well as with (ii) a 15% increase in GDF-15 levels over 6 months of follow-up (HR 1.68 [1.38–2.06] *p* < 0.001). Addition of change in GDF-15 to the fully adjusted model improved the C-statistics (*p* < 0.001). No interaction between treatment and baseline or change in GDF-15 on outcome was observed. GDF-15 was inversely associated with several indices of anemia and correlated positively with ferritin.

**Conclusions:**

In patients with HF and anemia, both higher baseline serum GDF-15 levels and an increase in GDF-15 during follow-up, were associated with worse clinical outcomes. GDF-15 did not identify subgroups of patients who might benefit from correction of anemia but was associated with several indices of anemia and iron status in the HF patients.

**Graphic abstract:**

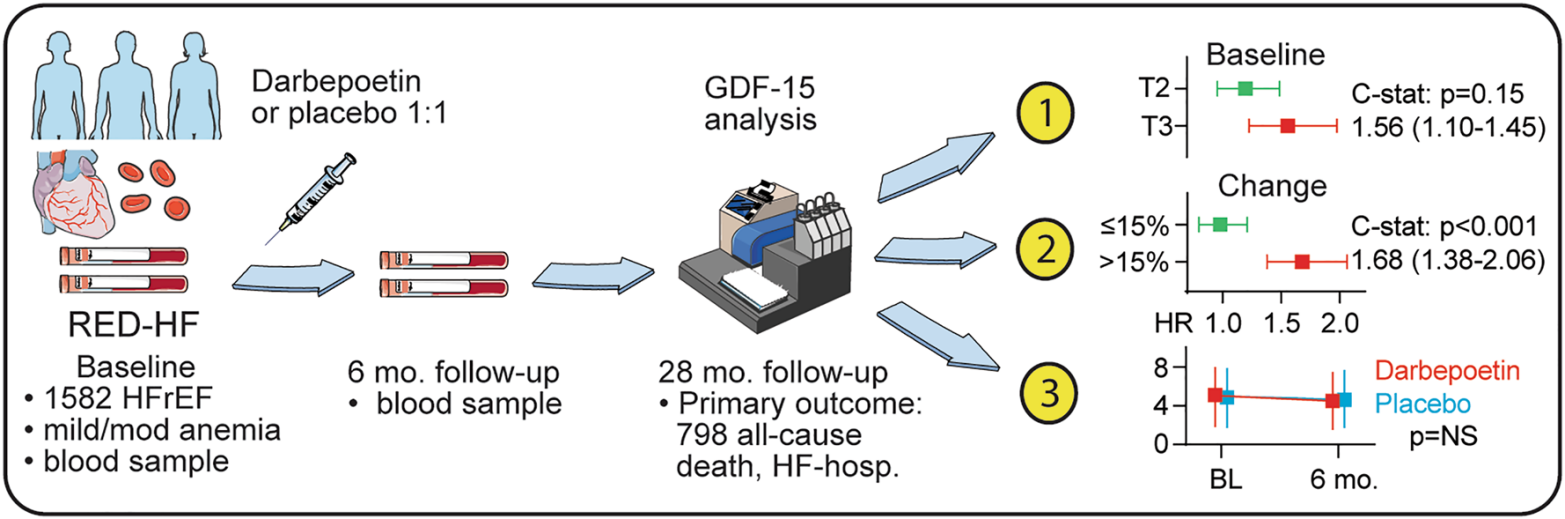

**Supplementary Information:**

The online version contains supplementary material available at 10.1007/s00392-021-01944-6.

## Introduction

Anemia is common in patients with heart failure (HF) and is associated with a high incidence of hospitalization and death [1–3]. The cause of anemia in patients with HF is often unknown, but may be related to iron deficiency or an absolute or relative deficiency of, or resistance to, erythropoietin as well as fluid retention [2–5]. Anemia in HF patients is associated with impaired renal function, potentially causing impaired erythropoietin production, and patients with HF often have systemic inflammation, which may lead to bone marrow suppression [2, 3].

Growth differentiation factor-15 (GDF-15) is a stress-responsive cytokine that is activated during inflammation and tissue remodeling The N-terminal propeptide is secreted upon proteolytic cleavage of the precursor protein as a disulfide-linked dimer with a molecular mass of ~ 28 kDa [6]. GDF-15 is expressed in cardiac tissue in patients with myocardial infarction [7] and in experimental models of pressure overload and cardiac hypertrophy, potentially contributing to myocardial remodeling [7, 8]. In clinical HF, serum or plasma levels of GDF-15 are increased and correlate with clinical and biochemical markers of disease severity [9]. Furthermore, high levels of GDF-15 are reported in patients with HF and anemia, correlated with iron status [10]. It has been suggested that GDF-15 may influence erythropoiesis by suppressing hepcidin expression, a major regulator of iron status [11]. However, the effect of GDF-1 on erythropoiesis is still unclear, and the suppressive effects of GDF-15 on hepcidin were tested in a very different setting (in vitro experiments in hepatocytes) than in HF patients [11]. Several studies suggest an association between high GDF-15 and adverse outcome in HF [12–15]. In the large Valsartan Heart Failure Trial (Val-HeFT, *n* = 1734), baseline GDF-15 was independently associated with mortality even after adjusting for multiple clinical and biochemical prognostic variables including BNP, *hs*-CRP, and *hs*-Troponin [16].

Personalized medicine is receiving increasing attention and there is a need to evaluate how promising biomarkers perform in more homogenous populations and to identify if they can predict a beneficial response to targeted therapy. Whereas use of the erythropoiesis-stimulating agent Darbepoetin was not associated with improvement in clinical outcomes in the overall population in RED-HF (Reduction of Events by Darbepoetin alfa in Heart Failure) trial [17], a benefit of correcting anemia in some subgroup of patients cannot be ruled out. Because GDF-15 is involved in inflammation and remodeling in cardiac and extracardiac tissues and may be related to erythropoiesis, we hypothesized that plasma concentration of GDF-15 may provide prognostic information in patients with HFrEF and anemia and identify patients who may benefit from darbepoetin alfa treatment. This hypothesis was tested in 1582 patients from the RED-HF trial who were followed for 28 months and with a primary outcome of the composite of death from any cause or first hospitalization for worsening of HF.

## Materials and methods

### Patients and study procedures

The study design and baseline characteristics of the RED-HF trial have been reported in detail previously [18, 19]. Patients were eligible for the study if they had the New York Heart Association (NYHA) functional class II–IV; HFrEF and left ventricular EF (LVEF) ≤ 40%; a hemoglobin level of 9.0–12.0 g per deciliter and were receiving guideline-recommended HF therapy. Patients were randomly assigned in a 1:1 ratio to receive either darbepoetin alfa or placebo. The study drug was administered subcutaneously, with doses adjusted according to hemoglobin level, which was measured in a blinded fashion.

### Study outcomes and definitions

The primary predefined outcome was a composite of death from any cause or first hospitalization for worsening of HF. The prespecified adjudicated secondary outcomes were (1) composite of death from cardiovascular (CV) causes or first hospitalization for worsening of HF, (2) death from any cause, and (3) CV death. Details on the definition and adjudication of all outcomes, with specific causes of CV death, have been described previously [17].

### Unresponsiveness to darbepoetin alfa

The hematopoietic response to DA was assessed as the percentage change in hemoglobin level between baseline and week 5 (after the 2 weight-based doses of DA) as previously reported [20]. Patients in the lowest quartile did not respond at all to DA and were considered non-responders, whereas subjects in the upper three quartiles were considered responders.

### Blood sampling and biochemical analyses

At randomization and 6-month follow-up, fasting venous blood was collected and serum and plasma were separated and stored at − 80 °C until thawing for assay. All blood samples were non-fasting and all biomarkers, except for GDF-15, were measured at a central laboratory including N-terminal pro-brain natriuretic peptide (NT-proBNP), high sensitivity assays for C-reactive protein (hsCRP), troponin T (hsTnT), serum iron, transferrin saturation and ferritin (measured as light-chain ferritin) (Medical Research Laboratories, Zaventem, Belgium). Plasma concentration of GDF-15 was analyzed by enzyme immunoassays from R&D Systems (Minneapolis, MN, USA) with intra- and inter-assay coefficients of variation < 10%. All samples were thawed an equal number of times (< 2 times). The analyses were performed in a 384-format using the combination of a SELMA (Jena, Germany) pipetting robot and a BioTek (Winooski, VT, USA) dispenser/washer. Absorption was read at 450 nm with wavelength correction set to 540 nm using an ELISA plate reader (Bio-Rad, Hercules, CA, USA).

### Statistical analysis

See Supplemental File for a full description of statistics. Kaplan–Meier curves were constructed to visualize and evaluate (log rank test) differences in survival. A restricted cubic spline analysis with three knots was undertaken on the primary outcome to assess linearity of risk. Survival analyses were performed using the Cox proportional hazard regression models to estimate hazard ratios (HRs) and 95% confidence intervals (CIs) for GDF-15 as a log-transformed continuous variables at baseline, which included age, gender, NYHA class, hospitalization for HF within 6 months, log serum creatinine, LVEF, etiology, body mass index (BMI), left bundle-branch block, history of atrial fibrillation or flutter, systolic blood pressure) at step one, log-transformed serum concentrations of NT-proBNP, hsTnT and hsCRP at step two. For the analysis of changes in GDF-15 concentrations from baseline to 6-month follow-up, a 15% relative change was used as cutoff, which is consistent with other studies [21]. Tertile changes were also assessed. A two-sided *p* value < 0.05 was considered to be significant. All statistical analyses were performed with the use of SAS software, version 9.2.

## Results

### Baseline correlates of GDF-15

Of the 2278 patients enrolled in the RED-HF study, baseline measurement of GDF-15 was available for 1582 (69%). The median plasma level of GDF-15 at baseline in the overall population was 4170 ng/L (IQR 2669–6272 ng/L). There were no differences in demographics comparing participants in the biomarker sub-study population with the main RED-HF population and few differences between the treatment groups except modestly higher NYHA class and platelet count in patients receiving Darbepoetin alfa (Supplemental Table 1). Table 1 shows the demographic and clinical characteristics according to tertiles of GDF-15. Elevated GDF-15 was associated with multiple characteristics indicating more severe HF (and worse outcomes) such as higher age, male sex, duration of disease, prevalence of diabetes, MI during the past 6 months, atrial fibrillation/flutter, poor kidney function, higher TnT and NT-proBNP levels and relevant for this population, lower iron, hemoglobin and transferrin saturation. As shown in Supplemental Table 2, stepwise linear regression identified lower hemoglobin as an independent predictor of GDF-15.

**Table 1:** Baseline characteristics of the patients by GDF-15 tertiles

Characteristic	T1, *n* = 529	T2, *n* = 526	T3, *n* = 527	*p *value
GDF-15 range (ng/L)	(500–3121)	(3122–5394)	(5399–20,480)	
Age, yrs	65 ± 13	71 ± 11	73 ± 10	< 0.001
Female sex	64	39	28	< 0.001
Race (white/black)	59/14	68/8	72/7	< 0.001
BMI (SD) kg/m^2^	27.6 ± 5.9	27.2 ± 5.7	26.4 ± 5.5	< 0.001
NYHA (III or IV)	64	67	68	0.304
Ischemic HF	60	75	80	< 0.001
Duration HF, yrs	5.0 ± 5.1	5.3 ± 5.4	5.8 ± 5.7	0.012
LVEF,%	31.1 ± 6.4	30.2 ± 6.8	29.5 ± 7.3	0.005
Medical history				
Hypertension	74	74	74	0.805
Diabetes	32	48	54	< 0.001
Atrial fibrillation or flutter	20	30	45	< 0.001
MI last 6 mo	28	35	47	< 0.001
Medication				
ACE or ARB	95	91	84	< 0.001
Beta-blocker	85	86	85	0.980
Diuretic	87	91	96	< 0.001
Systolic BP	123 ± 17	120 ± 18	117 ± 19	< 0.001
Heart rate, b.p.m.	73 ± 11	71 ± 12	73 ± 12	0.044
Biochemistry				
Creatinine, mg/dL	1.1 ± 0.4	1.5 ± 0.5	1.8 ± 0.6	< 0.001
eGFR, mL/min/1.73m^2^	64 ± 22	48 ± 19	39 ± 16	< 0.001
Hemoglobin, g/dL	11.2 ± 0.6	11.0 ± 0.7	10.9 ± 0.7	< 0.001
Transferrin saturation, %	27.5 ± 10.6	27.1 ± 10.8	26.4 ± 11.2	0.007
Iron, μg/dL	80.4 ± 34.5	75.7 ± 38.5	74.3 ± 37.3	0.019
Ferritin, μg/L	116 ± 133	165 ± 174	179 ± 190	< 0.001
Platelets, × 10^9^/L	251 ± 80	231 ± 80	212 ± 73	< 0.001
WBC, × 10^9^/L	6.5 ± 2.1	6.9 ± 2.2	6.7 ± 2.2	0.143
hsCRP, mg/dL	2.4 (1.1,5.4)	2.7 (1.1,7.2)	3.0 (1.3,7.2)	< 0.001
NT-proBNP, pmol/L	994 (220,2334)	1823 (762,3820)	2983 (1196,7002)	< 0.001
hsTnT, ng/ml	12 (9,17)	28 (21,34)	46 (33,71)	< 0.001

Patient characteristics are given as mean ± SD for continuous variables and % of cases for categorical variables

*ACE* angiotensin-converting enzyme, *ARB* angiotensin receptor blocker, *BMI* body mass index, *BP* blood pressure, *eGFR* estimated glomerular filtration rate, *hsCRP* high-sensitivity C-reactive protein, *hsTnT* high-sensitive troponin

### Association of baseline GDF-15 levels and outcomes

**Fig. 1:**
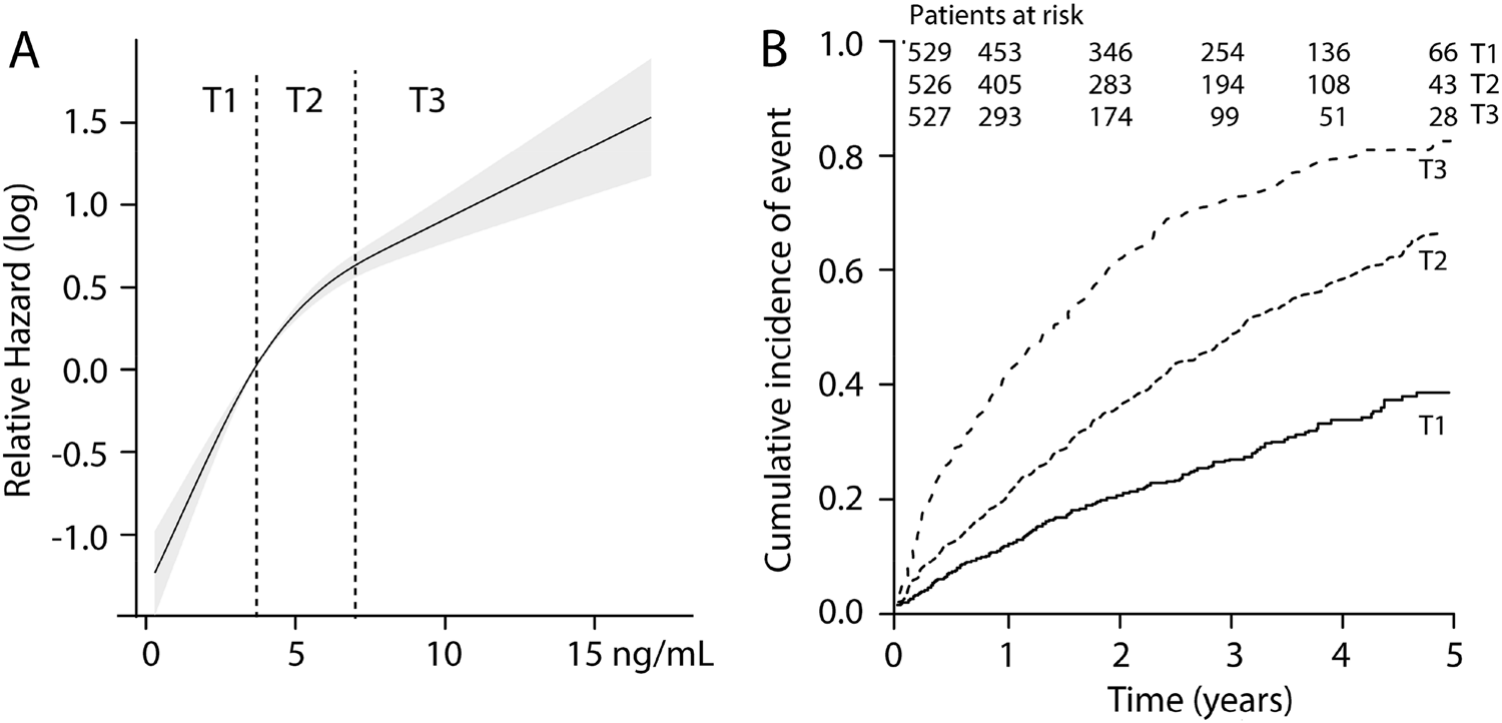
Association between baseline GDF-15 levels and the primary endpoint in the RED-HF cohort (*n* = 1582) during the whole study (mean follow-up 28 months, range 0.03–72.4 months) expressed as **A** restricted cubic spline with tertile cut-offs at enrollment shown as dotted lines and **B** Kaplan–Meier curves showing the cumulative incidence of the primary endpoint according to tertiles at enrollment

During a mean follow-up of 28 months (range 0.03–72.4 months), 798 patients reached a primary end-point, 716 patients reached the secondary endpoints, while 649 patients died and of these, 543 due to CV causes. Cubic spline analysis revealed an increase in risk of the primary endpoint with increasing GDF-15 (Fig. 1A) and this was mirrored by the Kaplan–Meier analysis (Fig. 1B) indicating a stepwise increase in the risk for the primary outcome with increasing tertiles (3rd tertile HR 4.05 [3.25–4.90]) relative to the lowest tertile in cox regression (adjusted for randomization) with a similar pattern for the other endpoints (Table 2). In multivariable analysis adjusting for pre-specified clinical variables (as outlined in statistical methods), the association between GDF-15 and outcome, evaluated according to tertiles and as a continuous variable, while attenuated, remained significantly associated with all end points with HR’s ranging from 2.47 to 2.62 for tertile 3 (all *p* < 0.001) (Step 1, Table 2). Addition of NT-proBNP, TnT and CRP to the multivariable models, attenuated the predictive value of GDF-15, but it remained significantly associated with all outcomes with HRs around 1.5 (Step 2, Table 2). Adding GDF-15 to the fully adjusted model did not improve the C-statistics; however, a significant effect on NRI was observed for all endpoints except cardiovascular mortality (Table 2).

**Table 2:** Association of baseline GDF-15 with outcomes

	Univariable	Step 1	Step 2	Δ C-index (*p* value)*	NRI (*p* value)*
All-cause mortality or first hospitalization for worsening heart failure, *n* = 798					
T2	2.10 (1.72‒2.57)	1.63 (1.32‒2.01)	1.20 (0.96‒1.49)		
T3	4.05 (3.35‒4.9)	2.57 (2.05‒3.23)	1.56 (1.23‒1.98)		
Continuous	2.29 (2.06‒2.54)	1.80 (1.58‒2.04)	1.26 (1.10‒1.45)		
p-trend/p-cont*	< 0.001/< 0.001	< 0.001/< 0.001	< 0.001/0.001	0.003 (0.148)	0.183 (< 0.001)
Cardiovascular mortality or first hospitalization for worsening heart failure, *n* = 716					
T2	2.10 (1.71‒2.59)	1.62 (1.30‒2.02)	1.20 (0.95‒1.50)		
T3	3.94 (3.22‒4.81)	2.47 (1.94‒3.13)	1.56 (1.23‒1.99)		
Continuous	2.25 (2.01‒2.51)	1.74 (1.52‒1.98)	1.22 (1.05‒1.41)		
p-trend/p-cont*	< 0.001/< 0.001	< 0.001/< 0.001	0.004/0.010	0.002 (0.212)	0.150 (0.003)
All-cause mortality, *n* = 649					
T2	2.22 (1.77‒2.78)	1.75 (1.39‒2.22)	1.32 (1.03‒1.68)		
T3	3.93 (3.17‒4.88)	2.62 (2.03‒3.38)	1.64 (1.25‒2.14)		
Continuous	2.32 (2.05‒2.59)	1.89 (1.64‒2.18)	1.37 (1.17‒1.60)		
p-trend/p-cont*	< 0.001/< 0.001	< 0.001/< 0.001	0.001/< 0.001	0.005 (0.108)	0.166 (0.001)
Cardiovascular mortality, *n* = 543					
T2	2.31 (1.81‒2.94)	1.82 (1.40‒2.35)	1.35 (1.04‒1.77)		
T3	3.75 (2.96‒4.74)	2.47 (1.86‒3.26)	1.50 (1.11‒2.01)		
Continuous	2.24 (1.97‒2.55)	1.82 (1.56‒2.13)	1.29 (1.08‒1.53)		
p-trend/p-cont	< 0.001/< 0.001	< 0.001/< 0.001	0.026/0.005	0.003 (0.220)	0.099 (0.061)

Hazard ratios and 95% confidence interval are shown for tertile 2 and 3 and for GDF-15 as a continuous (log) variable in univariate (UNI) analysis, when adjusted for clinical and biochemical variables (Step 1), and last for CRP, TnT and NT-proBNP (Step 2)

*Comparing the fully adjusted models with and without inclusion of log GDF15

### Comparison of the prognostic value of GDF-15 with NT-proBNP and hs-TnT

As shown in Supplemental Figure 1, GDF-15, NT-proBNP and TnT all contribute to a model without any other biomarker, with the biggest gain by NT-proBNP followed by TnT and GDF-15. Similarly, the largest decrease in c statistic was seen when NT-proBNP is subtracted from the full model including all biomarkers. For all-cause mortality, all markers contribute to a similar degree.

### Association of change in GDF-15 levels and outcomes

An increase in serum GDF-15 of > 15% during follow-up was associated with a higher incidence of the primary outcome following the second sampling (Table 3) adjusting for randomization (HR 1.39 [1.15–1.69] *p* < 0.002) and multivariable (HR 1.68 [1.38–2.06] *p* < 0.001) analyses with a similar pattern for the secondary composite and mortality outcomes (HR’s of 1.40–1.73 after full adjustment (Table 3). Furthermore, adding change in GDF-15 to the fully adjusted model improved the C-statistics (all *p* < 0.004) and a significant effect on NRI (all *p* < 0.001) was observed for all endpoints (Table 3).

**Table 3:** Association of change in GDF-15 with outcomes

	Univariable	Step 1	Step 2	Δ C-index (*p *value)*	NRI (*p *value)*
All-cause mortality or first hospitalization for worsening heart failure					
≤ − 15%	1.06 (0.86‒1.29)	0.95 (0.77‒1.17)	0.98 (0.80‒1.21)		
> 15%	1.39 (1.15‒1.69)	1.65 (1.36‒2.01)	1.68 (1.38‒2.06)		
p-trend	0.002	< 0.001	< 0.001	0.050 (< 0.001)	0.266 (< 0.001)
Cardiovascular mortality or first hospitalization for worsening heart failure					
≤ − 15%	1.09 (0.88‒1.35)	0.97 (0.78‒1.20)	1.00 (0.80‒1.25)		
> 15%	1.46 (1.19‒1.80)	1.72 (1.40‒2.11)	1.73 (1.40‒2.14)		
p-trend	0.001	< 0.001	< 0.001	0.051 (< 0.001)	0.228 (< 0.001)
All-cause mortality					
≤ − 15%	1.00 (0.80‒1.25)	0.98 (0.78‒1.24)	1.02 (0.81‒1.29)		
> 15%	1.23 (0.99‒1.53)	1.47 (1.18‒1.83)	1.40 (1.12‒1.76)		
p-trend	0.118	0.001	0.007	0.038 (0.004)	0.219 (< 0.001)
Cardiovascular mortality					
≤ − 15%	1.05 (0.82‒1.35)	1.03 (0.80‒1.33)	1.08 (0.84‒1.39)		
> 15%	1.35 (1.07‒1.71)	1.59 (1.25‒2.03)	1.49 (1.16‒1.91)		
p-trend	0.037	< 0.001	0.005	0.047 (0.002)	0.222 (< 0.001)

Hazard ratios and 95% confidence interval are shown for ≤ − 15% and > 15% change vs. no change (− 15‒15%) in univariate (UNI) analysis, when adjusted for clinical and biochemical variables (Step 1), and last for CRP, TnT and NT-proBNP (Step 2)

*Comparing the fully adjusted models with and without inclusion of log GDF15

**Fig. 2:**
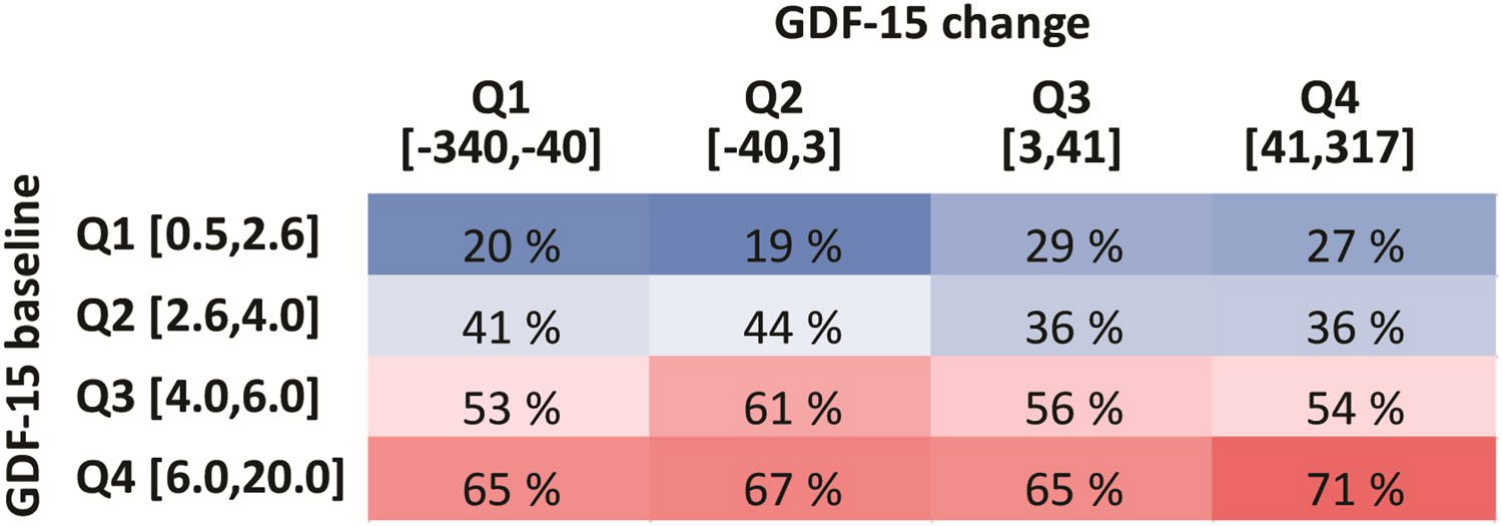
Heatmap showing association between baseline GDF-15 and change in GDF-15 on incidence (%) of the primary outcome. Both baseline (ng/mL) and change (%) are shown as quartiles with limits

Supplemental Table 3 shows the association between change in GDF-15 and outcomes evaluated as tertiles. This analysis supports that those with the largest increase in GFD-15 (T3) had a higher incidence of all outcome measures. The mid tertile (T2) displayed a lower risk compared to the lower tertile. Baseline GDF-15 levels were strongly correlated with levels at 6 months (*r* = 0.83, *p* < 0.001), but negatively correlated with change scores (*r* = − 0.23, *p* < 0.001). Finally, we made a heatmap of the incidence of the primary outcome according to quartiles of baseline and change in GDF-15. As shown in Fig. 2, the incidence was highest among all quartiles of change for quartile 4 of baseline GDF-15 with the highest incidence for quartile 4 of both baseline and change values. However, a markedly higher incidence for change in quartiles 3 and 4 vs. 1 and 2 was noted within quartile 1 of baseline GDF-15 (i.e. ~ 28% vs. ~ 20%).

### GDF-15 and iron metabolism

**Fig. 3:**
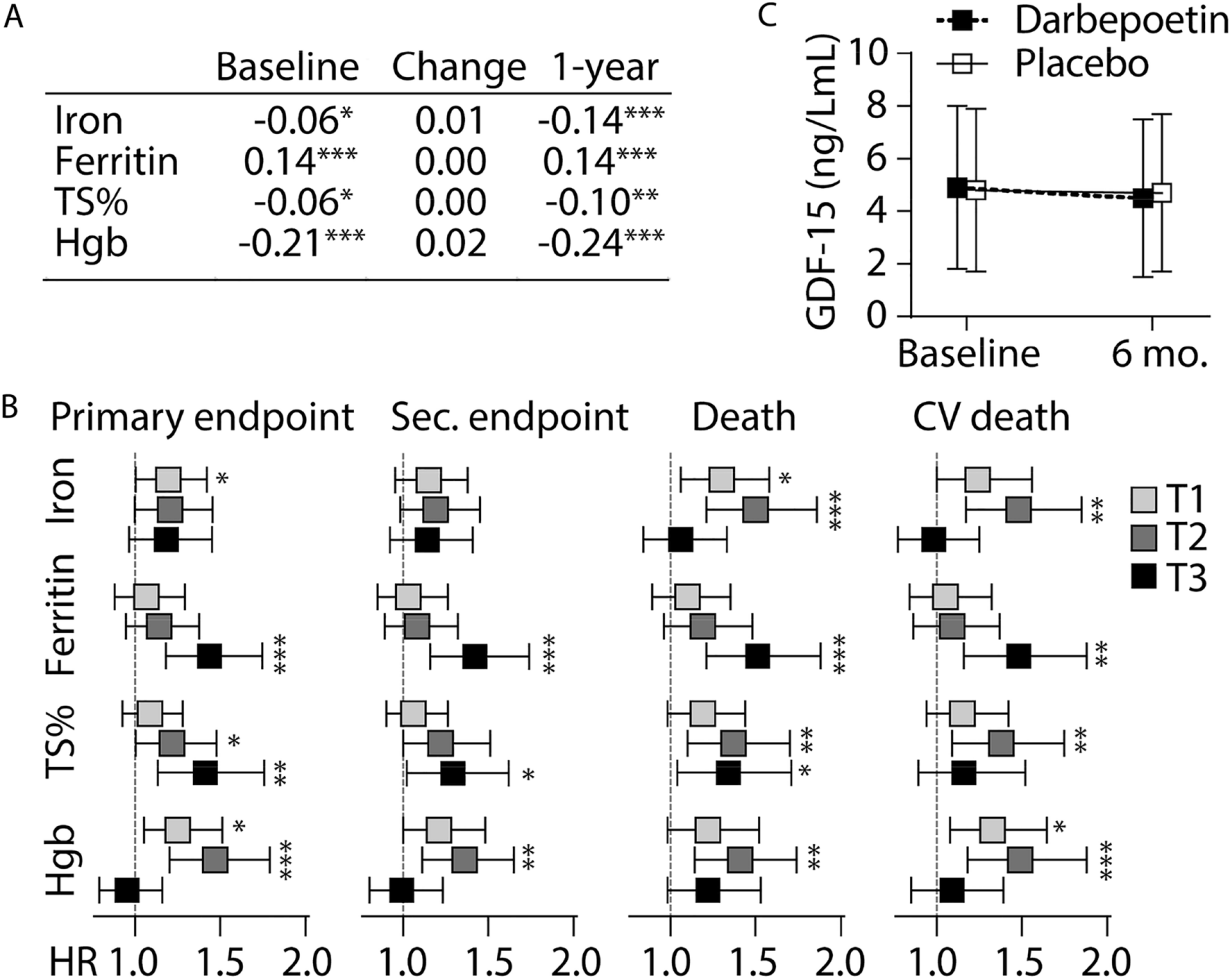
Association between GDF-15 and iron status in HF patients. **A** Correlation between GDF-15 and iron status markers at baseline and 1 year and change between these time-points. **B** Association between GDF-15 and the primary endpoint (death from any cause or first hospitalization for worsening of HF), secondary endpoint (death from cardiovascular causes or first hospitalization for worsening of HF), death and CV death within tertiles of iron status markers. The fully adjusted hazard ratio (HR) and 95% CI are shown. **C** Effect of Darbepoetin on GDF-15 levels in HF patients. *p < 0.05, **p < 0.01, *** < 0.001

To understand the potential role of GDF-15 in iron metabolism and erythropoiesis, we further analyzed associations between GDF-15 and different markers related to iron metabolism. As shown in supplemental Table 4 and Fig. 3A, at baseline GDF-15 was inversely correlated with hemoglobin and positively with ferritin, with weaker positive associations with transferrin saturation and iron. In the population as a whole, change in GDF-15 did not correlate with the iron parameters. At 6 months’ follow-up, somewhat stronger associations between GDF-15 and markers of iron metabolism were observed, with no significant differences according to treatment group.

### Interactions between iron deficiency markers, GDF-15 and outcomes

We next analyzed interactions between GDF-15 and iron status on outcome by dividing the iron status markers in tertiles and evaluating the prognostic role of GDF-15 (continuous log transformed) within each tertile. These data are presented adjusted for randomized treatment and after full multivariable adjustment in Supplemental Table 5 and graphically in Fig. 3B. The association between GDF15 and the primary outcome was not dependent on iron levels, while a stronger association was observed with increasing ferritin and transferrin saturation. Conversely, lower and mid-tertile levels of hemoglobin were associated with adverse outcomes with increasing GDF-15. These associations were quite similar for the secondary endpoints, but low or intermediate levels of iron were more strongly associated with worse outcome for the mortality endpoints (Supplemental Table 5).

### Effect of darbepoetin on GDF-15

Plasma GDF-15 levels were similar at baseline in the two treatment groups (mean ± SD 4866 ± 3123 ng/L vs*.* 4781 ± 3077 ng/L (*p* = 0.79), placebo and Darbepoetin, respectively. During the course of the study, a small decrease in GDF-15 was observed in the Darbepoetin group (*p* = 0.032, Fig. 3C), but not in the placebo group, yielding a modest but significant difference in relative change between the treatment groups (mean change 0.0 vs*.* 0.1 *p* = 0.039, placebo and Darbepoetin, respectively).

### Interactions between darbepoetin alpha treatment and GDF-15 and outcomes

We next evaluated whether baseline levels or change in GDF-15 could identify patients who could benefit from Darbepoetin treatment. We found no evidence for this for baseline GDF-15, with interaction *p* values for treatment*GDF-15 (tertiles) between 0.55 and 0.83 in unadjusted analysis for the different outcomes. A similar pattern was seen for change in GDF-15 (as categorized above) with interaction *p* values ranging between 0.11 and 0.82.

### GDF-15 and unresponsiveness to darbepoetin in HF patients

The median initial hemoglobin change in non-responders (*n* = 198) was − 0.25 g/dL and + 1.00 g/dL in the remainder of patients (*n* = 592). ROC analysis indicated no association between GDF-15 levels and unresponsiveness to ESA (AUC = 0.51). Evaluated as a continuous variable, GDF-15 did not identify non-responders: HR 0.99 (0.81–1.18) and the HRs were unmodified by the addition of markers of iron metabolism to the model (i.e. ferritin, hemoglobin, transferrin saturation, iron). Similar results were obtained when GDF-15 was evaluated according to tertiles:tertile 2 HR 0.86 (95% CI: 0.54–1.35); tertile 3 HR 0.85 (0.53–1.34). These HRs were unmodified by the addition of markers of iron metabolism to the model.

## Discussion

In our study, higher baseline GDF-15 levels or an increase in GDF-15 levels during follow-up were associated with poor prognosis in adjusted analysis including TnT, CRP and NT-proBNP, all strong predictors of outcome in HF patients [22, 23]. Furthermore, change in GDF-15 improved the discrimination and the magnitude of net improvement in sensitivity and specificity when added to these models. GDF-15 was associated with all iron status markers with the strongest correlation with hemoglobin but could not identify unresponsiveness or responsiveness to Darbepoetin. Our findings further support a role for GDF-15 as a strong and independent prognostic marker in patients with HF.

GDF-15 has been reported to add prognostic information in several CV disorders including myocardial infarction (MI), atherosclerosis, aortic stenosis, pulmonary hypertension and ischemic stroke [6, 24] as well as in HFrEF [12–16, 25] and more recently also in HF with preserved EF (HFpEF) [13, 15, 25]. However, like several other inflammatory markers, GDF-15 is not specific for HF or other CV disorders and is not useful as a diagnostic tool, although capable of giving prognostic information as we have shown. Kempf et al. showed that in 455 patients with systolic HF, GDF-15 predicted total mortality independent of biochemical and clinical variables including NT-proBNP [12]. Chan et al. found that in 730 patients with systolic HF, baseline GDF-15 was associated with the composite outcome of all-cause mortality and first re-hospitalization of HF also after adjusting for established clinical and biochemical variables including TnT and NT-proBNP, with a similar pattern in HFpEF [13]. Gaggin et al. found that in 151 patients with chronic HF, GDF-15 together with TnT and the soluble version of the interleukin 1 receptor member ST2 was independently associated with CV events also after adjusting for NT-proBNP [14]. In a substudy of Val-HeFT (*n* = 1734), baseline GDF-15 levels were associated with all-cause mortality and the first morbid event after adjusting for clinical and biochemical variables [16]; the association remained significant for all-cause mortality but not for the first morbid event when further adjustments were made for TnT, CRP and NT-proBNP. Similar to the Val-HeFT study, we found that an increase in GDF-15 was strongly associated with poor prognosis, with significant improvement in discrimination analysis for all outcomes. However, the negative correlation between baseline GDF-15 and change in GDF-15, and relatively larger increase in incidence of the primary outcome with increasing quartiles of change within quartile 1 of baseline GDF-15, suggests patients with the largest increase are not only those presenting with high GDF-15 levels. Indeed, thus, serial analysis could be beneficial in identifying patients not detected by the initial measurement.

The independent association between GDF-15 and adverse outcome in chronic HF may have several non-mutually exclusive explanations. First, GDF-15 is strongly up-regulated in the myocardium during wall stress and ischemia [7, 8], and it is possible that the plasma levels in HFrEF at least partly reflect up-regulation of GDF-15 in failing myocardium. Second, GDF-15 seems to reflect activation of inflammatory, myocardial remodeling and apoptotic pathways-, and the ability of a measure like GDF-15 to mirror several pathological processes is an important feature of a robust biomarker. Notably, GDF-15 exhibits potent anti-inflammatory, anti-hypertrophic and anti-apoptotic properties and its up-regulation during HF seems to reflect multiple and counteractive mechanisms [7, 8].

While the RED-HF trial failed to detect a benefit of an erythropoiesis-stimulating agent on clinical outcomes, this does not exclude the possibility that some individuals may benefit from correction of anemia. Enhanced hepcidin production has been implicated in the pathogenesis of anemia in chronic inflammatory disorders like HF [26], and GDF-15 was recently identified as a hepcidin-suppression factor [11]. However, the suppressive effect of GDF-15 on hepcidin was not tested in this study and has not been reported in HF patients in other studies. Nor has an interaction between GDF-15 and erythropoiesis been clarified to our knowledge. In the present study, we found mostly weak correlations between GDF-15 and markers of iron metabolism. However, a stronger association between GDF15 and the primary outcome was observed with increasing ferritin and transferrin saturation, and conversely, lower and mid-tertile levels of hemoglobin were associated with adverse outcomes with increasing GDF-15, suggesting some interaction between GDF-15 and iron metabolism. Moreover, GDF-15 levels in our study population seems to be higher than in previous study in HF patients [9]. Although the GDF-15 assay was not calibrated against a universal standard, it is possible that the additional burden of anemia could have contributed to the elevated levels GDF-15 levels in the present study as compared to HF patients without anemia as also has been suggested by others [10].

## Limitations

The strengths of the present investigation include the large number of patients studied with a high event rate, longitudinal sampling and adjustment for multiple relevant confounders. On the other hand, a randomized trial may not necessarily reflect the “real-world” HF population and the use of composite endpoints has an inborn limitation. Our patients also had anemia, and our results will, therefore, not apply to all chronic HF cohorts. However, anemia is common in HF, in particular in advanced cases. Finally, the lack of data on hepcidin levels is also a limitation of the present study.

## Conclusions

Our findings indicate that GDF-15 is a promising prognostic marker in patients with chronic HF and anemia, with correlation to indices of iron metabolism. Future studies should examine whether this marker could be used for treatment-related decision-making and risk stratification in these patients.

## Supplementary Information

Below is the link to the electronic supplementary material.

Supplementary file1 (DOCX 137 kb)

## Data Availability

The data that support the findings of this study are available from Amgen but restrictions apply to the availability of these data, which were used under license for the current study, and so are not publicly available. Data are, however, available from the authors upon reasonable request and with permission of Amgen.

## References

[1] Anand I, McMurray JJ, Whitmore J, Warren M, Pham A, McCamish MA, Burton PB (2004). Anemia and its relationship to clinical outcome in heart failure. Circulation.

[2] O'Meara E, Murphy C, McMurray JJ (2004). Anemia and heart failure. Curr Heart Fail Rep.

[3] Anand IS, Gupta P (2018). Anemia and iron deficiency in heart failure: current concepts and emerging therapies. Circulation.

[4] Westenbrink BD, Visser FW, Voors AA, Smilde TD, Lipsic E, Navis G, Hillege HL, van Gilst WH, van Veldhuisen DJ (2007). Anaemia in chronic heart failure is not only related to impaired renal perfusion and blunted erythropoietin production, but to fluid retention as well. Eur Heart J.

[5] von Haehling S, Ebner N, Evertz R, Ponikowski P, Anker SD (2019). Iron deficiency in heart failure: an overview. JACC Heart Fail.

[6] Wollert KC, Kempf T (2012). Growth differentiation factor 15 in heart failure: an update. Curr Heart Fail Rep.

[7] Kempf T, Eden M, Strelau J, Naguib M, Willenbockel C, Tongers J, Heineke J, Kotlarz D, Xu J, Molkentin JD, Niessen HW, Drexler H, Wollert KC (2006). The transforming growth factor-beta superfamily member growth-differentiation factor-15 protects the heart from ischemia/reperfusion injury. Circ Res.

[8] Xu J, Kimball TR, Lorenz JN, Brown DA, Bauskin AR, Klevitsky R, Hewett TE, Breit SN, Molkentin JD (2006). GDF15/MIC-1 functions as a protective and antihypertrophic factor released from the myocardium in association with SMAD protein activation. Circ Res.

[9] Wollert KC, Kempf T, Wallentin L (2017). Growth differentiation factor 15 as a biomarker in cardiovascular disease. Clin Chem.

[10] Przybylowski P, Wasilewski G, Bachorzewska-Gajewska H, Golabek K, Dobrzycki S, Malyszko J (2014). Growth differentiation factor 15 is related to anemia and iron metabolism in heart allograft recipients and patients with chronic heart failure. Transpl Proc.

[11] Tanno T, Bhanu NV, Oneal PA, Goh SH, Staker P, Lee YT, Moroney JW, Reed CH, Luban NL, Wang RH, Eling TE, Childs R, Ganz T, Leitman SF, Fucharoen S, Miller JL (2007). High levels of GDF15 in thalassemia suppress expression of the iron regulatory protein hepcidin. Nat Med.

[12] Kempf T, von Haehling S, Peter T, Allhoff T, Cicoira M, Doehner W, Ponikowski P, Filippatos GS, Rozentryt P, Drexler H, Anker SD, Wollert KC (2007). Prognostic utility of growth differentiation factor-15 in patients with chronic heart failure. J Am Coll Cardiol.

[13] Chan MM, Santhanakrishnan R, Chong JP, Chen Z, Tai BC, Liew OW, Ng TP, Ling LH, Sim D, Leong KT, Yeo PS, Ong HY, Jaufeerally F, Wong RC, Chai P, Low AF, Richards AM, Lam CS (2016). Growth differentiation factor 15 in heart failure with preserved vs. reduced ejection fraction. Eur J Heart Fail.

[14] Gaggin HK, Szymonifka J, Bhardwaj A, Belcher A, De Berardinis B, Motiwala S, Wang TJ, Januzzi JL (2014). Head-to-head comparison of serial soluble ST2, growth differentiation factor-15, and highly-sensitive troponin T measurements in patients with chronic heart failure. JACC Heart Fail.

[15] George M, Jena A, Srivatsan V, Muthukumar R, Dhandapani VE (2016). GDF 15—a novel biomarker in the offing for heart failure. Curr Cardiol Rev.

[16] Anand IS, Kempf T, Rector TS, Tapken H, Allhoff T, Jantzen F, Kuskowski M, Cohn JN, Drexler H, Wollert KC (2010). Serial measurement of growth-differentiation factor-15 in heart failure: relation to disease severity and prognosis in the Valsartan Heart Failure Trial. Circulation.

[17] Swedberg K, Young JB, Anand IS, Cheng S, Desai AS, Diaz R, Maggioni AP, McMurray JJ, O'Connor C, Pfeffer MA, Solomon SD, Sun Y, Tendera M, van Veldhuisen DJ, Committees R-H, Investigators R-H (2013). Treatment of anemia with darbepoetin alfa in systolic heart failure. N Engl J Med.

[18] McMurray JJ, Anand IS, Diaz R, Maggioni AP, O'Connor C, Pfeffer MA, Polu KR, Solomon SD, Sun Y, Swedberg K, Tendera M, van Veldhuisen DJ, Wasserman SM, Young JB, Committees R-H, Investigators (2009). Design of the reduction of events with Darbepoetin alfa in heart failure (RED-HF): a phase III, anaemia correction, morbidity-mortality trial. Eur J Heart Fail.

[19] McMurray JJ, Anand IS, Diaz R, Maggioni AP, O'Connor C, Pfeffer MA, Solomon SD, Tendera M, van Veldhuisen DJ, Albizem M, Cheng S, Scarlata D, Swedberg K, Young JB, Investigators R-HC (2013). Baseline characteristics of patients in the reduction of events with Darbepoetin alfa in heart failure trial (RED-HF). Eur J Heart Fail.

[20] van der Meer P, Grote Beverborg N, Pfeffer MA, Olson K, Anand IS, Westenbrink BD, McMurray JJV, Swedberg K, Young JB, Solomon SD, van Veldhuisen DJ (2018). Hyporesponsiveness to Darbepoetin Alfa in patients with heart failure and anemia in the RED-HF study (reduction of events by Darbepoetin Alfa in heart failure): clinical and prognostic associations. Circ Heart Fail.

[21] Masson S, Anand I, Favero C, Barlera S, Vago T, Bertocchi F, Maggioni AP, Tavazzi L, Tognoni G, Cohn JN, Latini R, Valsartan Heart Failure T, Gruppo Italiano per lo Studio della Sopravvivenza nell'Insufficienza Cardiaca-Heart Failure I (2012). Serial measurement of cardiac troponin T using a highly sensitive assay in patients with chronic heart failure: data from 2 large randomized clinical trials. Circulation.

[22] Ferreira JP, Ouwerkerk W, Tromp J, Ng L, Dickstein K, Anker S, Filippatos G, Cleland JG, Metra M, van Veldhuisen DJ, Voors AA, Zannad F (2020). Cardiovascular and non-cardiovascular death distinction: the utility of troponin beyond N-terminal pro-B-type natriuretic peptide. Findings from the BIOSTAT-CHF study. Eur J Heart Fail.

[23] Pellicori P, Zhang J, Cuthbert J, Urbinati A, Shah P, Kazmi S, Clark AL, Cleland JGF (2020). High-sensitivity C-reactive protein in chronic heart failure: patient characteristics, phenotypes, and mode of death. Cardiovasc Res.

[24] Xu X, Li Z, Gao W (2011). Growth differentiation factor 15 in cardiovascular diseases: from bench to bedside. Biomarkers.

[25] Izumiya Y, Hanatani S, Kimura Y, Takashio S, Yamamoto E, Kusaka H, Tokitsu T, Rokutanda T, Araki S, Tsujita K, Tanaka T, Yamamuro M, Kojima S, Tayama S, Kaikita K, Hokimoto S, Ogawa H (2014). Growth differentiation factor-15 is a useful prognostic marker in patients with heart failure with preserved ejection fraction. Can J Cardiol.

[26] van der Putten K, Jie KE, van den Broek D, Kraaijenhagen RJ, Laarakkers C, Swinkels DW, Braam B, Gaillard CA (2010). Hepcidin-25 is a marker of the response rather than resistance to exogenous erythropoietin in chronic kidney disease/chronic heart failure patients. Eur J Heart Fail.

