# Supplementary material for: Growth differentiation factor 15 predicts poor prognosis in patients with heart failure and reduced ejection fraction and anemia: results from RED-HF
Source: Clin Res Cardiol. 2021 Oct 5;111(4):440–50. doi: 10.1007/s00392-021-01944-6 (PMC8971146; doi:10.1007/s00392-021-01944-6)
Supplement: Supplementary file 1 — Supplementary file1 (DOCX 137 kb) [file 392_2021_1944_MOESM1_ESM.docx]

**Supplemental file** “Growth differentiation factor 15 predicts poor prognosis in heart failure patients with anemia-results from RED-HF”

Ueland et al.

**Methods**

**Statistics**

Trends across tertiles of GDF-15 were tested using Kruskal-Wallis H test, one-way ANOVA or chi-square depending on distribution and variable type (i.e. categorical or continuous). For comparing treatment effects on the GDF-15, the Mann-Whitney U test was used on change values while Wilcoxon matched pairs test was used to assess longitudinal changes within groups. Stepwise linear regression was used to identify the most important predictors of GDF-15. Kaplan-Meier curves were constructed to visualize and evaluate (log rank test) differences in survival. A restricted cubic spline analysis with three knots was undertaken on the primary outcome to assess linearity of risk. Survival analyses were performed using the Cox proportional hazard regression models to estimate hazard ratios (HRs) and 95% confidence intervals (CIs) for GDF-15 as a log-transformed continuous variables at baseline, which included mainly clinical variables at step one in addition to randomization (age, gender, NYHA class, hospitalization for HF within 6 months, log serum creatinine, LVEF, etiology, body mass index (BMI), left bundle-branch block, history of atrial fibrillation or flutter, systolic blood pressure). At step two, log-transformed serum concentrations of NT-proBNP, hsTnT and hsCRP were included. The adjustment model was prespecified. For the analysis of changes in GDF-15 concentrations from baseline to 6-month follow-up, a 15% relative change was used as cutoff, which is consistent with other studies (1). Harrel’s C-statistic and Net reclassification improvement (NRI) was calculated to evaluate the prognostic usefulness of biomarkers. We also compared the prognostic value of GDF-15 with NT-proBNP and hs-TnT in two ways: First, we evaluated C-statistics for the addition of GDF-15, NT-proBNP and TnT to a model including adjustment variables from step 1 as described above but including CRP. Second, we evaluated C-statistics the exclusion of GDF-15, NT-proBNP and TnT from the full model including all adjustment variables and GDF-15, NT-proBNP and TnT. Associations between GDF-15 and indices of iron deficiency were assessed by spearman correlation. Interactions between iron deficiency markers, GDF-15 and outcomes were evaluated by dividing the iron deficiency markers in tertiles and evaluating the association between GDF-15 and outcomes within each tertile in unadjusted and adjusted analysis as described above. ROC analysis and logistic regression was used to evaluate the association between GDF-15 levels and unresponsiveness to Darbepoetin alfa. A two-sided p-value <0.05 was considered to be significant. All statistical analyses were performed with the use of SAS software, version 9.2.

1. Masson S, Anand I, Favero C et al. Serial measurement of cardiac troponin T using a highly sensitive assay in patients with chronic heart failure: data from 2 large randomized clinical trials. Circulation 2012;125:280-8.

Supplementary Table 1. Comparison between participants in the biomarker sub-study population with the main RED-HF population.

| **Characteristic** | **Main trial** | **Sub-study** | **P-value** | **Placebo**  **N=792** | **Darbepoetin alfa**  **N=790** | **P-value** |
| --- | --- | --- | --- | --- | --- | --- |
|  | (n=2278) | (n=1582) |  | (n=792) | (n=790) |  |
| Age, yrs. | 70±11 | 70±12 | 0.468 | 69±12 | 70±12 | 0.371 |
| Female sex | 41 | 43 | 0.250 | 44 | 42 | 0.202 |
| Race (white/black) | 68/9 | 66/10 | 0.423 | 66/11 | 67/9 | 0.231 |
| BMI (SD) kg/m^2^ | 27.2±5.7 | 27.1±5.7 | 0.446 | 27±6 | 27±6 | 0.891 |
| NYHA (III or IV) | 65 | 66 | 0.456 | 64 | 69 | 0.036 |
| Ischemic HF | 73 | 72 | 0.353 | 71 | 72 | 0.842 |
| Duration HF, yrs. | 5.3±5.3 | 5.4±5.4 | 0.759 | 5±5 | 5±5 | 0.456 |
| LVEF,% | 30.3±6.8 | 30.3±6.8 | 1.000 | 30±7 | 30±7 | 0.698 |
| **Medical history** |  |  |  |  |  |  |
| Hypertension | 74 | 74 | 0.733 | 73 | 75 | 0.546 |
| Diabetes | 46 | 45 | 0.339 | 44 | 45 | 0.758 |
| Atrial fibrillation or flutter | 33 | 32 | 0.463 | 32 | 31 | 0.720 |
| MI last 6 mo. | 37 | 37 | 0.862 | 34 | 39 | 0.082 |
| **Medication** |  |  |  |  |  |  |
| ACE or ARB | 89 | 90 | 0.216 | 89 | 92 | 0.034 |
| Beta-blocker | 85 | 85 | 0.965 | 86 | 84 | 0.450 |
| Diuretic | 92 | 91 | 0.930 | 91 | 92 | 0.237 |
| Systolic BP | 120±18 | 120±18 | 0.889 | 121±18 | 119±18 | 0.103 |
| Heart rate, bpm. | 72±11 | 72±11 | 0.635 | 72±11 | 72±11 | 0.418 |
| **Biochemistry** |  |  |  |  |  |  |
| Creatinine, mg/dL | 1.5±0.6 | 1.5±0.6 | 0.451 | 1.5±0.6 | 1.5±0.6 | 0.703 |
| eGFR, mL/min/1.73m^2^ | 50±21 | 50±22 | 0.546 | 50±22 | 50±22 | 0.853 |
| Hemoglobin, g/dL | 11.0±0.7 | 11.0±0.7 | 0.699 | 11.0±0.7 | 11.0±0.7 | 0.560 |
| Transferrin saturation, % | 26.8 ±10.8 | 27.9±10.9 | 0.735 | 27.3±11.6 | 26.7±10.2 | 0.254 |
| Iron, μg/dL | NA |  | NA | 75.7±34.4 | 78.0±39.2 | 0.227 |
| Ferritin, μg/L | NA |  | NA | 149±153 | 158±184 | 0.267 |
| Platelets, x10^9^/L | 232±79 | 232±79 | 0.842 | 228±78 | 236±81 | 0.050 |
| WBC, x10^9^/L | 6.9±2.2 | 6.7±2.2 | 0.534 | 6.7±2.2 | 6.8±2.1 | 0.467 |
| hsCRP, mg/dL | 2.7 (0.8,6.3) | 2.7 (0.8,6.6) | 0.444 | 2.6 [0.7, 6.5 | 2.7 [0.8, 6.8] | 0.122 |
| NT-proBNP, pmol/L | 1823 (653,4244) | 1840 (592,4209) | 0.986 | 1832 [628,4189] | 1850 [557,4231] | 0.869 |
| hsTnT, ng/ml | 26 (15,43) | 26 (15,41) | 0.826 | 25 [15,41] | 27 [16,41] | 0.383 |

Patient characteristics are given as mean ± SD for continuous variables and % of cases for categorical variables. ACE, angiotensin-converting enzyme; ARB, angiotensin receptor blocker; BMI, body mass index; BP, blood pressure; eGFR, estimated glomerular filtration rate; hsCRP, high-sensitivity C-reactive protein; hsTnT, high-sensitive troponin.

**Supplemental Table 2.** Baseline predictors of GDF-15

|  | Coefficients (95% CI) | P value |
| --- | --- | --- |
| (Intercept) | 7.48 (5.06‒9.92) | <0.001 |
| hsTnT (Log), ng/mL | 0.53 (0.36‒0.69) | <0.001 |
| Creatinine, mg/dL | 1.36 (1.11‒1.62) | <0.001 |
| NT-proBNP (Log), pmol/L | 0.63 (0.47‒0.78) | <0.001 |
| Hemoglobin, g/dL | -0.47 (-0.64‒-0.3) | <0.001 |
| Systolic BP | -0.02 (-0.02‒-0.01) | <0.001 |
| Age, yrs. | 0.03 (0.02‒0.04) | <0.001 |
| History of Diabetes | 0.48 (0.23‒0.72) | <0.001 |
| MI last 6 mo. | 0.44 (0.19‒0.69) | <0.001 |
| Platelets | 0.00 (-0.01‒0.00) | <0.001 |
| Heart rate, bpm. | 0.02 (0.01‒0.03) | <0.001 |
| hsCRP (Log), mg/dL | 0.19 (0.06‒0.32) | 0.004 |
| Race, other | 0.66 (0.22‒1.11) | 0.005 |
| History of Atrial fibrillation or flutter | 0.27 (-0.04‒0.58) | 0.004 |
| Diuretic | -0.50 (-0.92‒-0.08) | 0.040 |

**Supplemental Table 3.** Association of Change in GDF-15 with Outcomes evaluated as tertiles.

|  | Univariable | Step 1 | Step 2 |
| --- | --- | --- | --- |
| **All-Cause Mortality or First Hospitalization for Worsening Heart Failure** | | | |
| T2 | 0.62 (0.50‒0.76) | 0.86 (0.69‒1.07) | 0.83 (0.66‒1.04) |
| T3 | 1.32 (1.09‒1.59) | 1.62 (1.33‒1.96) | 1.58 (1.30‒1.92) |
| p-trend | <0.001 | <0.001 | <0.001 |
|  |  |  |  |
| **Cardiovascular Mortality or First Hospitalization for Worsening Heart Failure** | | | |
| T2 | 0.57 (0.46‒0.72) | 0.81 (0.64‒1.03) | 0.78 (0.61‒1.00) |
| T3 | 1.32 (1.08‒1.60) | 1.62 (1.32‒1.98) | 1.57 (1.28‒1.93) |
| p-trend | <0.001 | <0.001 | <0.001 |
|  |  |  |  |
| **All-Cause Mortality** | | | |
| T2 | 0.61 (0.48‒0.78) | 0.79 (0.62‒1.02) | 0.77 (0.59‒0.98) |
| T3 | 1.21 (0.98‒1.49) | 1.34 (1.08‒1.66) | 1.28 (1.02‒1.58) |
| p-trend | <0.001 | <0.001 | <0.001 |
|  |  |  |  |
| **Cardiovascular Mortality** | | | |
| T2 | 0.59 (0.45‒0.77) | 0.76 (0.57‒1.00) | 0.74 (0.55‒0.97) |
| T3 | 1.25 (0.99‒1.58) | 1.39 (1.1‒1.76) | 1.31 (1.03‒1.65) |
| p-trend | <0.001 | <0.001 | <0.001 |

Hazard ratios and 95% confidence interval are shown for T2 change (-420 – 340 ng/L) and T3 change (>340 ng/mL) vs. T1 change (<420 ng/L) in univariate (UNI) analysis, when adjusted for clinical and biochemical variables (step 1), and lastly for CRP, TnT and NT-proBNP (Step 2). * comparing the fully adjusted models with and without inclusion of log GDF15.

**Supplemental Table 4.** Correlation (Spearman) between baseline, change and 6 month levels of GDF-15 and markers of iron deficiency and anemia in the RED-HF cohort.

|  |  |  | Change |  | 6 months | | |
| --- | --- | --- | --- | --- | --- | --- | --- |
|  | Baseline | All | Placebo | Darb. | All | Placebo | Darb. |
| Iron | -0.06* | 0.01 | 0.01 | -0.02 | -0.14*** | -0.11** | -0.15*** |
| Ferritin | 0.14*** | 0.00 | -0.02 | 0.03 | 0.14*** | 0.19*** | 0.11** |
| Transferrin | -0.06* | 0.00 | 0.00 | 0.01 | -0.10** | -0.07 | -0.11** |
| Hemoglobin | -0.21*** | 0.02 | -0.02 | -0.02 | -0.24*** | -0.30*** | -0.19*** |

*p<0.05, *p<0.01, ***p<0.001

**Supplemental Table 5.** Interactions between GDF-15 and iron status on outcome.

|  | Univariate* | Full adjustement** |
| --- | --- | --- |
|  | HR (95% CI) p-value | HR (95% CI) p-value |
| **All-Cause Mortality or First Hospitalization for Worsening Heart Failure** | | |
| Iron <60 μg/dL | 1.90 (1.67 - 2.17) <0.001 | 1.19 (1.00 - 1.42) 0.046 |
| Iron 60-81 μg/dL | 1.86 (1.61 - 2.14) <0.001 | 1.20 (1.00 - 1.45) 0.055 |
| Iron >81 μg/dL | 1.81 (1.57 - 2.09) <0.001 | 1.18 (0.96 - 1.45) 0.108 |
| Ferritin <65 μg/L | 1.56 (1.36 - 1.78) <0.001 | 1.07 (0.88 - 1.29) 0.517 |
| Ferritin 65-149 μg/L | 1.94 (1.69 - 2.23) <0.001 | 1.14 (0.95 - 1.38) 0.17 |
| Ferritin >149 μg/L | 2.19 (1.90 - 2.52) <0.001 | 1.44 (1.18 - 1.75) <0.001 |
| Transferrin saturation <21% | 1.75 (1.55 - 1.98) <0.001 | 1.09 (0.92 - 1.28) 0.311 |
| Transferrin saturation 21-29% | 1.86 (1.62 - 2.14) <0.001 | 1.22 (1.00 - 1.48) 0.049 |
| Transferrin saturation >29% | 2.06 (1.75 - 2.41) <0.001 | 1.41 (1.13 - 1.76) 0.002 |
| Hemoglobin <10.8 g/dL | 1.94 (1.70 - 2.23) <0.001 | 1.20 (0.99 - 1.45) 0.059 |
| Hemoglobin 10.8-11.45 g/dL | 2.13 (1.85 - 2.46) <0.001 | 1.34 (1.11 - 1.61) 0.002 |
| Hemoglobin > 11.45 g/dL | 1.59 (1.38 - 1.85) <0.001 | 1.07 (0.87 - 1.31) 0.52 |
| **Cardiovascular Mortality or First Hospitalization for Worsening Heart Failure** | | |
| Iron <60 μg/dL | 1.89 (1.64 - 2.17) <0.001 | 1.15 (0.95 - 1.38) 0.145 |
| Iron 60-81 μg/dL | 1.84 (1.59 - 2.13) <0.001 | 1.19 (0.98 - 1.45) 0.082 |
| Iron >81 μg/dL | 1.77 (1.52 - 2.05) <0.001 | 1.14 (0.92 - 1.41) 0.234 |
| Ferritin <65 μg/L | 1.53 (1.33 - 1.75) <0.001 | 1.03 (0.85 - 1.26) 0.759 |
| Ferritin 65-149 μg/L | 1.90 (1.64 - 2.20) <0.001 | 1.08 (0.89 - 1.32) 0.439 |
| Ferritin >149 μg/L | 2.19 (1.88 - 2.55) <0.001 | 1.42 (1.16 - 1.74) 0.001 |
| Transferrin saturation <21% | 1.75 (1.54 - 1.99) <0.001 | 1.06 (0.90 - 1.26) 0.479 |
| Transferrin saturation 21-29% | 1.83 (1.58 - 2.13) <0.001 | 1.22 (1.00 - 1.51) 0.054 |
| Transferrin saturation >29% | 1.95 (1.65 - 2.30) <0.001 | 1.29 (1.02 - 1.62) 0.031 |
| Hemoglobin <10.8 g/dL | 1.97 (1.68 - 2.29) <0.001 | 1.21 (1.00 - 1.48) 0.052 |
| Hemoglobin 10.8-11.45 g/dL | 2.03 (1.75 - 2.36) <0.001 | 1.36 (1.11 - 1.65) 0.003 |
| Hemoglobin > 11.45 g/dL | 1.60 (1.38 - 1.85) <0.001 | 0.99 (0.80 - 1.23) 0.931 |
| **Death** |  |  |
| Iron <60 μg/dL | 1.95 (1.68 - 2.27) <0.001 | 1.30 (1.06 - 1.58) 0.011 |
| Iron 60-81 μg/dL | 2.06 (1.75 - 2.42) <0.001 | 1.50 (1.21 - 1.86) <0.001 |
| Iron >81 μg/dL | 1.63 (1.39 - 1.90) <0.001 | 1.06 (0.84 - 1.33) 0.616 |
| Ferritin <65 μg/L | 1.59 (1.36 - 1.85) <0.001 | 1.10 (0.89 - 1.35) 0.396 |
| Ferritin 65-149 μg/L | 1.96 (1.67 - 2.30) <0.001 | 1.19 (0.96 - 1.48) 0.106 |
| Ferritin >149 μg/L | 2.14 (1.83 - 2.50) <0.001 | 1.51 (1.21 - 1.88) <0.001 |
| Transferrin saturation <21% | 1.85 (1.61 - 2.13) <0.001 | 1.19 (0.98 - 1.44) 0.075 |
| Transferrin saturation 21-29% | 1.87 (1.60 - 2.20) <0.001 | 1.37 (1.10 - 1.70) 0.004 |
| Transferrin saturation >29% | 1.91 (1.61 - 2.26) <0.001 | 1.34 (1.04 - 1.71) 0.021 |
| Hemoglobin <10.8 g/dL | 1.96 (1.68 - 2.29) <0.001 | 1.22 (0.98 - 1.52) 0.071 |
| Hemoglobin 10.8-11.45 g/dL | 2.17 (1.85 - 2.54) <0.001 | 1.41 (1.14 - 1.74) 0.002 |
| Hemoglobin > 11.45 g/dL | 1.55 (1.32 - 1.81) <0.001 | 1.22 (0.98 - 1.53) 0.074 |
| **CV death** |  |  |
| Iron <60 μg/dL | 1.93 (1.63 - 2.27) <0.001 | 1.24 (1.00 - 1.56) 0.055 |
| Iron 60-81 μg/dL | 2.08 (1.74 - 2.47) <0.001 | 1.48 (1.17 - 1.85) 0.001 |
| Iron >81 μg/dL | 1.55 (1.31 - 1.83) <0.001 | 0.98 (0.77 - 1.25) 0.878 |
| Ferritin <65 μg/L | 1.55 (1.31 - 1.83) <0.001 | 1.05 (0.84 - 1.32) 0.678 |
| Ferritin 65-149 μg/L | 1.89 (1.59 - 2.25) <0.001 | 1.09 (0.86 - 1.37) 0.49 |
| Ferritin >149 μg/L | 2.14 (1.81 - 2.54) <0.001 | 1.48 (1.16 - 1.88) 0.002 |
| Transferrin saturation <21% | 1.87 (1.60 - 2.18) <0.001 | 1.15 (0.94 - 1.42) 0.177 |
| Transferrin saturation 21-29% | 1.82 (1.53 - 2.17) <0.001 | 1.38 (1.09 - 1.75) 0.008 |
| Transferrin saturation >29% | 1.79 (1.49 - 2.15) <0.001 | 1.16 (0.89 - 1.52) 0.26 |
| Hemoglobin <10.8 g/dL | 2.06 (1.73 - 2.45) <0.001 | 1.18 (0.93 - 1.50) 0.163 |
| Hemoglobin 10.8-11.45 g/dL | 2.03 (1.71 - 2.42) <0.001 | 1.49 (1.18 - 1.88) 0.001 |
| Hemoglobin > 11.45 g/dL | 1.54 (1.30 - 1.81) <0.001 | 1.09 (0.85 - 1.39) 0.486 |

*adjusting for randomized treatment, ** Full multivariable adjustment (age, gender, NYHA class, hospitalization for HF within 6 months, log serum creatinine, LVEF, etiology, body mass index, left bundle-branch block, history of atrial fibrillation or flutter, systolic blood pressure, log-transformed serum concentrations of NT-proBNP, hsTnT and hsCRP.

***
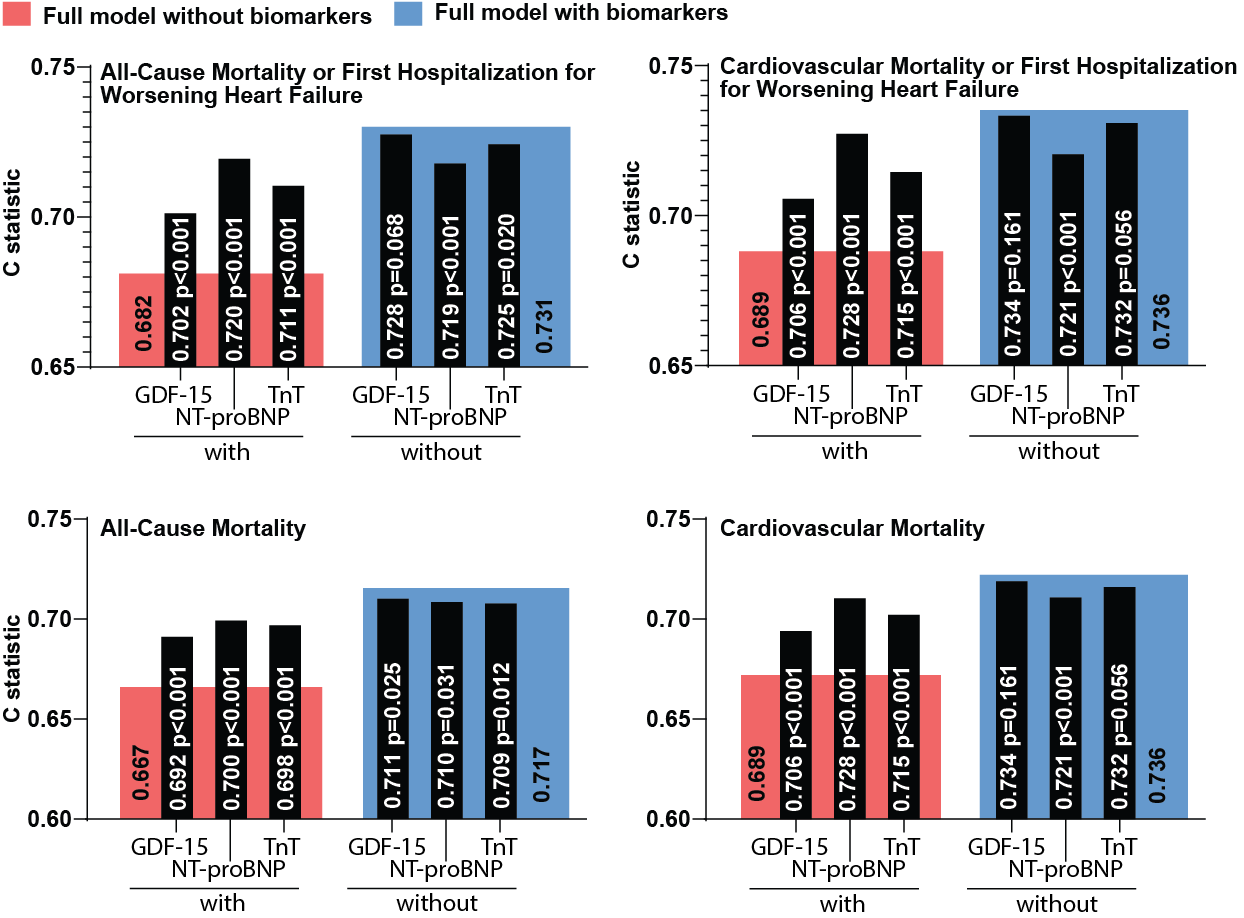
***

**Supplemental Figure 1.** Comparison of the prognostic value of GDF-15, NT-proBNP and TnT on outcomes in RED-HF. The graphs show c-statistics for GDF-15, NT-proBNP and TnT in two ways: on the left side of each graph, we show the c statistics for the addition of each biomarker on a model including all adjustment variables as described under statistics and CRP in addition (marked in pink) but excluding GDF-15, NT-proBNP and TnT and on the right side, the exclusion of GDF-15, NT-proBNP and TnT from the full model including all adjustment variables and GDF-15, NT-proBNP and TnT (marked in blue). Data given in graphs are the c statistic and corresponding p-value
